# Molecular and Supramolecular Structures of Triiodides and Polyiodobismuthates of Phenylenediammonium and Its N,N-dimethyl Derivative

**DOI:** 10.3390/molecules26185712

**Published:** 2021-09-21

**Authors:** Tatiana A. Shestimerova, Nikita A. Golubev, Mikhail A. Bykov, Andrei V. Mironov, Sergey A. Fateev, Alexey B. Tarasov, Ivan Turkevych, Zheng Wei, Evgeny V. Dikarev, Andrei V. Shevelkov

**Affiliations:** 1 Department of Chemistry, Lomonosov Moscow State University, 119991 Moscow, Russia; shestimerova@inorg.chem.msu.ru (T.A.S.); nikgol98@yandex.ru (N.A.G.); mich.bykov@gmail.com (M.A.B.); avmironov@inorg348-1.chem.msu.ru (A.V.M.); alexey.bor.tarasov@gmail.com (A.B.T.); 2 Department of Materials Science, Lomonosov Moscow State University, 119991 Moscow, Russia; saf1al@yandex.ru; 3 Sensing System Research Center, National Institute of Advanced Industrial Science and Technology, Tsukuba 305-8565, Japan; ivan.turkevych@aist.go.jp; 4 Department of Chemistry, University at Albany, SUNY, Albany, NY 12222, USA; zwei@albany.edu (Z.W.); edikarev@albany.edu (E.V.D.)

**Keywords:** triiodides, hybrid organic–inorganic compounds, phenylenediammonium, bismuth, supramolecular ensemble, molecular structure, intermolecular interactions

## Abstract

Despite remarkable progress in photoconversion efficiency, the toxicity of lead-based hybrid perovskites remains an important issue hindering their applications in consumer optoelectronic devices, such as solar cells, LED displays, and photodetectors. For that reason, lead-free metal halide complexes have attracted great attention as alternative optoelectronic materials. In this work, we demonstrate that reactions of two aromatic diamines with iodine in hydroiodic acid produced phenylenediammonium (PDA) and N,N-dimethyl-phenylenediammonium (DMPDA) triiodides, PDA(I_3_)_2_⋅2H_2_O and DMPDA(I_3_)I, respectively. If the source of bismuth was added, they were converted into previously reported PDA(BiI_4_)_2_⋅I_2_ and new (DMPDA)_2_(BiI_6_)(I_3_)⋅2H_2_O, having band gaps of 1.45 and 1.7 eV, respectively, which are in the optimal range for efficient solar light absorbers. All four compounds presented organic–inorganic hybrids, whose supramolecular structures were based on a variety of intermolecular forces, including (N)H⋅⋅⋅I and (N)H⋅⋅⋅O hydrogen bonds as well as I⋅⋅⋅I secondary and weak interactions. Details of their molecular and supramolecular structures are discussed based on single-crystal X-ray diffraction data, thermal analysis, and Raman and optical spectroscopy.

## 1. Introduction

Polyiodides have recently reemerged as attentively studied compounds, as they can provide reaction media for the fabrication of absorber layers for hybrid perovskite solar cells [1,2]. The desire to create efficient synthetic pathways for production of hybrid perovskites is a strong motivation for finding alternatives to solvent-assisted fabrication methods, which exhibit various drawbacks such as inaccurate coverage of the perovskite layer across the substrate. In contrast, the polyiodide-assisted fabrication method is capable of transforming a layer of metallic lead several tens of nanometers thick into a uniform layer of a hybrid perovskite by applying triiodide of the selected organic and/or inorganic cation [3,4]. Although Pb-based hybrid perovskites retain their advantages for solar power stations located in non-residential areas, the neurotoxicity of lead should be considered extremely seriously when building integrated perovskite PV systems. For that reason, there is an incentive to develop lead-free halometallates as potential replacements for lead-containing perovskites. Unfortunately, hybrid perovskites obtained by isovalent substitutions with group 14 elements (Sn and Ge) are extremely unstable. In recent years, many metals other than lead have been tested as building centers of various metal–halide complexes, leading to remarkable progress in understanding how their dimensionality affects optical absorption and carrier dynamics [5,6,7,8,9]. Moreover, it has been demonstrated that among multifarious halometallates, there exists a smaller family of polyhalometallates. It was shown that the inclusion of I_2_ molecules or I_3_^−^ anions can lead to narrower band gaps and better optical properties in terms of potential applications as light-harvesting compounds in perovskite-like solar cells [10,11,12,13,14].

Although the history of polyiodides can be traced back to the 18th century, and many reviews have been published on different topics related to this family of compounds, for example [15,16,17,18], various aspects of their chemistry remain unknown; most notably, phase relations for low-melting polyiodides of different organic compounds and mechanisms of their transformation into polyiodometallates are poorly investigated [2,3,10,12]. Some other issues, including intramolecular bonding in polyiodides, require further investigation [13,15]. In the context of perovskite solar cells, several important questions have come to the forefront. First of all, it is desired to scrutinize the chemistry of low-melting polyiodides of strong organic cations; secondly, the ability of various metals to form hybrid compounds composed of iodometallates and additional I_2_ molecules or polyiodide anions by facile reactions needs to be examined; finally, the role of various weak bonds in assembling compounds and affecting their optical properties is expected to be studied in detail.

In this paper, we present new triiodides of rigid organic dications: phenylenediammonium (PDA) and its N,N-dimethyl derivatives (DMPDA), PDA(I_3_)_2_⋅2H_2_O (**1**) and DMPDA(I_3_)I (**2**), respectively. We examined their crystal and molecular structures, paying attention to non-covalent bonds assembling cationic and anionic moieties into supramolecular architectures. We also discuss two polyiodobismuthates, PDA(BiI_4_)_2_⋅I_2_ (**3**) [14] as previously reported and (DMPDA)_2_(BiI_6_)(I_3_)⋅2H_2_O (**4**) which was prepared for the first time, formed by the same cations, in which either I_2_ molecules or I_3_^-^ anions serve as additional building units. Details of molecular and supramolecular structures of those compounds are described.

## 2. Results

Two new triiodides **1** and **2** were synthesized by facile reactions of the respective diamine or its hydrochloride with 57% aqueous HI. Natural oxidation of concentrated hydroiodic acid in air under visible light was the source of molecular diiodine, which could easily convert into I_3_^-^ anions in the acidic medium [17]. Compound **1** appeared as large black crystals sensitive to moist air. In a nitrogen atmosphere it began to decompose at near 310 K, losing one I_2_ molecule per formula, whereas the second I_2_ molecule was released near 380 K along with water. Compound **2** formed brown crystals, which showed good stability in moist air up to 387 K; upon further heating it decomposed releasing one I_2_ molecule per formula (see Figures S1 and S2 of Supporting Information).

Upon using BiI_3_ as the source of bismuth, two Bi-containing polyiodide derivatives of PDA and DMPDA were synthesized. Compound **3** has been previously reported elsewhere [14]; it was crystallized from the mixture of BiI_3_ and PDA in concentrated HI and shown to be stable up to 373 K. For the synthesis of **4**, BiI_3_ was reacted with the solution of DMPDA hydrochloride in concentrated HI followed by slow evaporation, leading to the crystallization of dark red crystals. The compound was moderately stable in air and showed signs of discoloration within several days of storage. Upon heating in a nitrogen flow it began to decompose at 380 K, releasing some I_2_ and crystallization water (see Figure S3 of Supporting Information).

**Figure 1:**
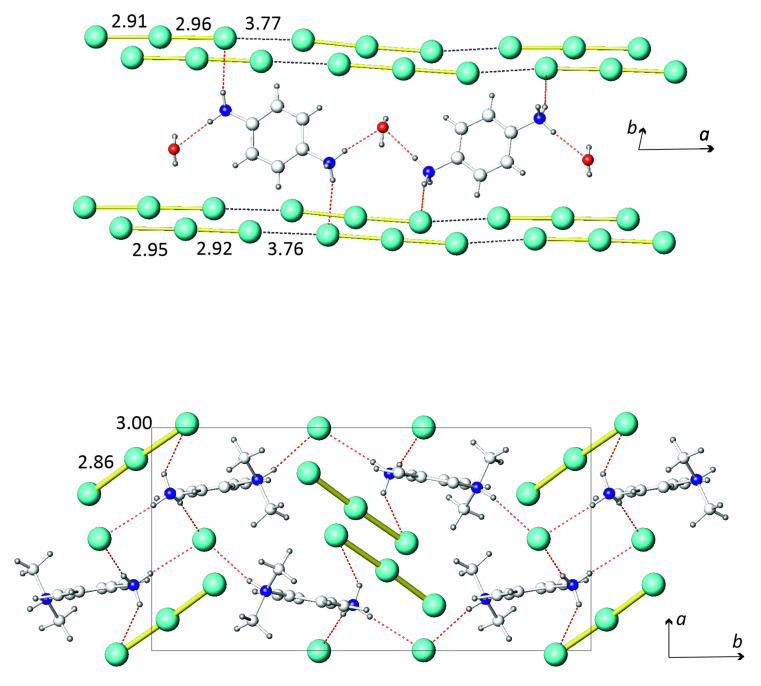
Crystal structures of **1** (**top**) and **2** (**bottom**). Iodine, green; oxygen, red; nitrogen, blue; carbon, light gray; hydrogen, dark gray. Hydrogen (N)H⋅⋅⋅O and (N)H⋅⋅⋅I bonds are shown as red dashed lines and I⋅⋅⋅I bonds as black dashed lines. The I–I and I⋅⋅⋅I interatomic distances are given in Å.

The molecular structures of **1** and **2** contained similar I_3_^−^ anions. The structure of **1** showed clear segregation of anionic and cationic substructures. The former consisted of I_3_^-^ anions (Figure 1) that formed chains running along the *a* axis of the orthorhombic unit cell. There were two independent chains that showed slightly different geometry of the I–I interatomic distances. In both types of chains, the I_3_^−^ anions were asymmetric, with the shorter and longer I–I bond distances being 2.91 and 2.96 Å, respectively, in one chain, and 2.92 and 2.95 Å in another. Despite the slight difference, the average I–I bonding distance fell within the range of 2.92–2.95 Å, typical for I_3_^-^ anions [15,18]. Both I_3_^−^ anions exhibited slight deviations from linearity, the I–I–I angle being 177 deg. Within both chains, the triiodide anions were assembled in a head-to-tail fashion with I⋅⋅⋅I interanion separations of 3.76–3.77 Å. These distances were much shorter than the corresponding van der Waals contacts of 3.9–4.3 Å [15], but were longer than the I⋅⋅⋅I separations of 3.50 Å in crystalline diiodine, which ensured the assembly of I_2_ molecules in a three-dimensional structure [17]. We also noted that the head-to-tail stacking of various polyiodide units was quite typical for the chemistry of iodine, and the observed range of the I⋅⋅⋅I distances covered the range of 3.55-3.85 Å [19,20,21,22,23], which accommodates the values found in **1** (Table 1).

The cationic part of **1** was comprised of PDA dications and water molecules. These moieties were linked by classic (N)H⋅⋅⋅O hydrogen bonds and thus formed zigzag chains running along the *a* axis (Figure 1). The (N)H⋅⋅⋅O bond had O⋅⋅⋅H separations of 1.99–2.03 Å and N-H⋅⋅⋅O angles of 171–172 deg., which is typical for this type of hydrogen bond. The links between the cationic and anionic parts were provided by (N)H⋅⋅⋅I bonds of 2.75 Å (Table 2). Comparing these bond distances with the literature data shows that they were slightly shorter than the typical (N)H⋅⋅⋅I bonds of 2.85–2.87 Å [24,25].

The crystal structure of **2** comprised three building units: the I^-^ and I_3_^-^ anions and the DMPDA dications (Figure 1). The triiodide anion was considerably asymmetric, and the I–I distances were 2.86 and 3.00 Å, which corresponds to the average I–I distance of 2.93 Å, typical for triiodide anions irrespective of their actual symmetry [15,18]. The I_3_^−^ anion was linked to the DMPDA cation through a sole (N)H⋅⋅⋅I interaction of 2.79 Å. The I_3_^−^ anions were well separated from each other; no I⋅⋅⋅I intermolecular bonds were present. Another anion in the crystal structure of **2** was I^−^, which formed no covalent bonds but was linked to three DMPDA cations by (N)H⋅⋅⋅I bonds of 2.54, 2.60, and 2.63 Å. Although such bond lengths are significantly shorter than typical (2.85–2.87 Å), they were previously reported for several compounds [26]. Notably, the I^-^ anion formed three intermolecular bonds; such a mode of supramolecular coordination was reported for several crystal structures featuring weak (N)H⋅⋅⋅I bonds. A combination of (N)H⋅⋅⋅I bonds between DMPDA cations and I_3_^-^ and I^-^ anions led to the three-dimensional crystal structure of **2**.

**Figure 2:**
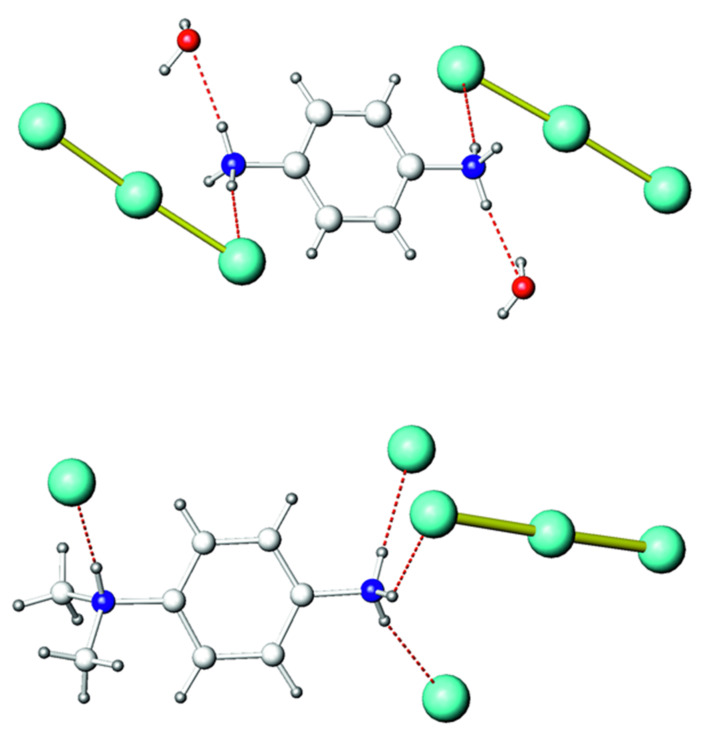
Local coordination of diammonium cations in the crystal structures of **1** (**top**) and **2** (**bottom**). Iodine, green; oxygen, red; nitrogen, blue; carbon, light gray; hydrogen, dark gray. Hydrogen (N)H⋅⋅⋅O and (N)H⋅⋅⋅I bonds are shown as red dashed lines.

While the molecular structures of **1** and **2** featured congenial PDA and DMPDA cations, their linking modes were somewhat different. PDA used two hydrogen atoms of each NH_3_ group to form hydrogen bonds. There was one (N)H⋅⋅⋅I and one (N)H⋅⋅⋅O bond on each side of PDA, whereas the third hydrogen atom of each NH_3_ group remained unused in bonding (Figure 2). By contrast, the DMPDA cation used all of its available hydrogen atoms attached to nitrogens to form four hydrogen bonds.

**Figure 3:**
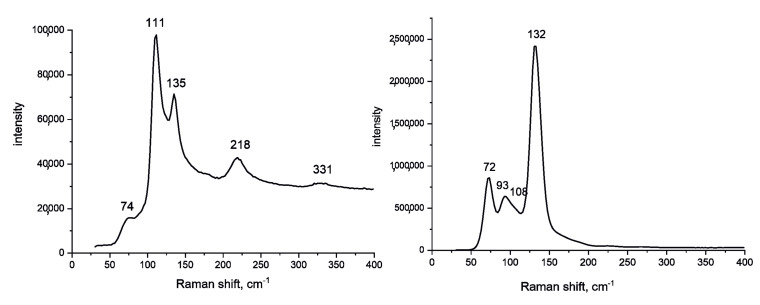
Raman spectra of **1** (**left**) and **2** (**right**).

The (N)H⋅⋅⋅I bonds caused an asymmetry of the I_3_^-^ anions. Indeed, in both structures triiodide anions formed only one (N)H⋅⋅⋅I bond with the organic cation, such that the I–I bond involving the iodine atom that formed the (N)H⋅⋅⋅I bond was longer than the I–I bond to the iodine atom with no other neighbors. This also caused a slight deviation of the I_3_^−^ anions from linearity (Table 2). The asymmetry of the triiodide anion was manifested in the Raman spectra of **1** and **2** (Figure 3). In **1**, I_3_^−^ was only slightly asymmetric. As a consequence, the highest band was observed at 111 cm^−1^ and was supplemented by two overtones at 218 and 331 cm^−1^. This band is typical for all triiodides with an average I–I bond length of 2.92–2.94 Å, and can be ascribed to the symmetric stretching of the anion [27,28,29,30]. The band at 135 cm^−1^ should be assigned to the doubly degenerate asymmetric stretching of I_3_^-^ [29]. This band is forbidden by selection rules if I_3_^-^ conforms to the *D_ih_* symmetry. However, if the symmetry is lower, this mode becomes active in the Raman spectrum, with its intensity growing as the deviation from the *D_ih_* symmetry increases. The bands at lower Raman shifts were difficult to assign because various modes can appear in this region, including asymmetric bending of I_3_^−^, vibrations of (N)H⋅⋅⋅I bonds, and even lattice vibrations. Most likely, the band at 74 cm^−1^ can be ascribed to the doubly degenerate bending of the asymmetric I_3_^−^ anion [26].

The Raman spectrum of **2** was substantially different. It exhibited the most intensive band at 132 cm^−1^ assigned to the asymmetric stretching of I_3_^−^. This reflects the higher degree of deviation from the *D_ih_* symmetry compared to **1**. Indeed, the difference between two I–I distances in I_3_^−^ in the structure of **2** was 0.14 Å compared to only 0.05 Å in **1**.

The molecular structures of compounds **3** and **4** both featured [BiI_6_] octahedra as principal building units. Compound **3** was reported in our earlier work [14], and hereafter we refer to it only to provide a comparison with the new compound **4**.

Compounds **3** and **4** appeared as black and dark red crystals, respectively. Compound **3** showed remarkable stability at ambient conditions; it could be stored in a Petri dish for weeks without any sign of degradation. In contrast, **4** slowly decomposed in open air, and discoloration became visible after a few days of storage. Upon heating, both compounds started to decompose at 373 (**3**) and 386 (**4**) K, with releasing of I_2_ (**3**) or I_2_ and crystallization water (**4**) at the first step (see Figure S3 of Supporting Information).

**Figure 4:**
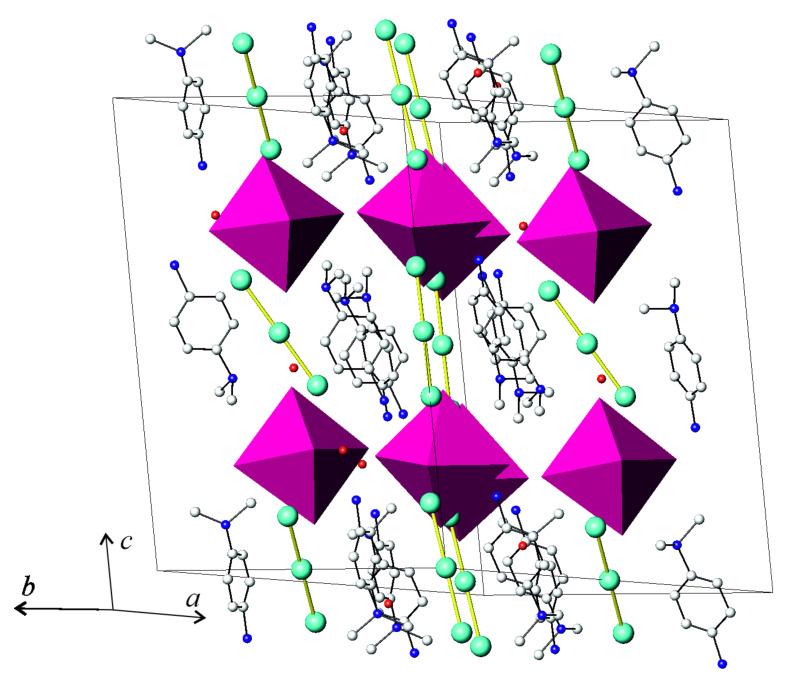
View of the crystal structure of **4**. Iodine, green; oxygen, red; nitrogen, blue; carbon, light gray; (BiI_6_)^3-^ octahedra, purple. Hydrogen atoms are omitted for clarity.

The molecular structure of **4** consisted of four building blocks: (BiI_6_)^3−^ octahedra, I_3_^−^ anions, DMPDA cations, and water molecules (Figure 4). The (BiI_6_)^3−^ octahedra were slightly irregular, with six Bi–I bond distances ranging from 3.01 to 3.19 Å and I–Bi–I angles only slightly deviating from 90 or 180 deg (Table 1). This geometry is typical for stand-alone (BiI_6_)^3−^ octahedra [31,32,33]. Each of two independent I_3_^-^ anions in the structure of **4** was symmetric (*D_ih_*); the I–I bond lengths were 2.92 Å in one anion and 2.95 Å in the other. DMPDA cations and crystallization water composed the cationic part. They were linked by hydrogen bonds to each other and to iodine atoms of the (BiI_6_)^3−^ and I_3_^−^ anions. This kind of arrangement of (BiI_6_)^3−^ and I_3_^−^ anions was previously reported in the literature [11,12]; its general motif is bridging of organic cations by a triiodide anion with the help of hydrogen bonds.

**Figure 5:**
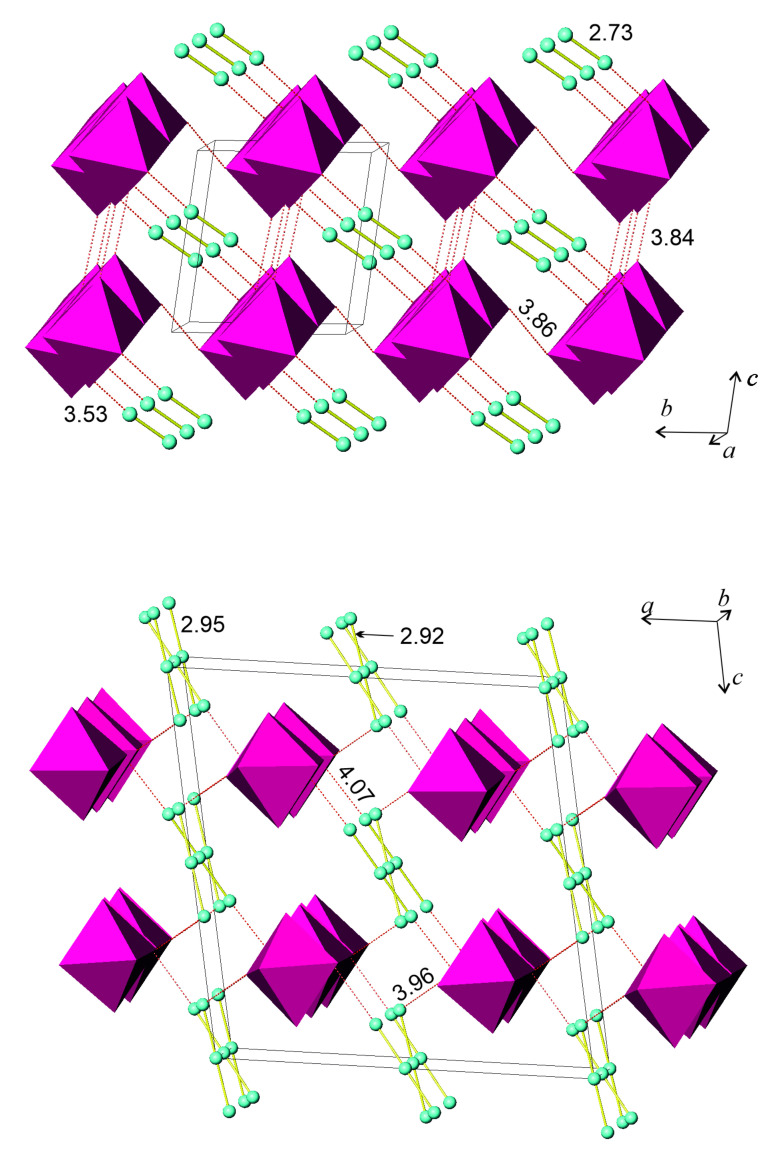
Patterns of I–I covalent (yellow, solid) and I⋅⋅⋅I intermolecular (red, dashed) interactions in the crystal structures of **3** (**top**) and **4** (**bottom**). Only iodine atoms (green) and [BiI_6_] octahedra (purple) are shown. Interatomic distances are given in Å.

Comparing the crystal structures of **3** and **4**, it appears clear that despite both being polyiodobismuthates and having similar constitution, their structures were significantly different in details. First of all, they differed in the nature and role of the polyiodide moiety. In the structure of **3**, I_2_ molecules with the d(I–I) = 2.72 Å covalent bond linked together the [BiI_6_] octahedra of the [BiI_4_]^1−^ chain-like anion with so-called secondary bonds of 3.53 Å in length [15], whereas **4** contained loosely bound I_3_^−^ anions. At the same time, both structures had a number of weak I⋅⋅⋅I interactions between iodine atoms in the vertices of the [BiI_6_] octahedra in **3**, and between the (BiI_6_)^3−^ stand-alone octahedra and I_3_^-^ anions in **4** (Figure 5). In the former structure, the I⋅⋅⋅I distances of 3.84–3.86 Å point to noncovalent interactions, which are found in many other iodobismuthates [34,35,36]. Although their energy is estimated to be 5–25 kJ⋅mole^−1^, they are known to serve as an additional factor in increasing the thermodynamic stability of a compound [35,37] and are capable of diminishing the band gap [24,36]. Conversely, the I⋅⋅⋅I distances of 3.96–4.07 Å in **4** are very close to the twofold van der Waals radius of iodine, which, according to different sources, is defined as 1.95–2.15 Å [38,39,40].

**Figure 6:**
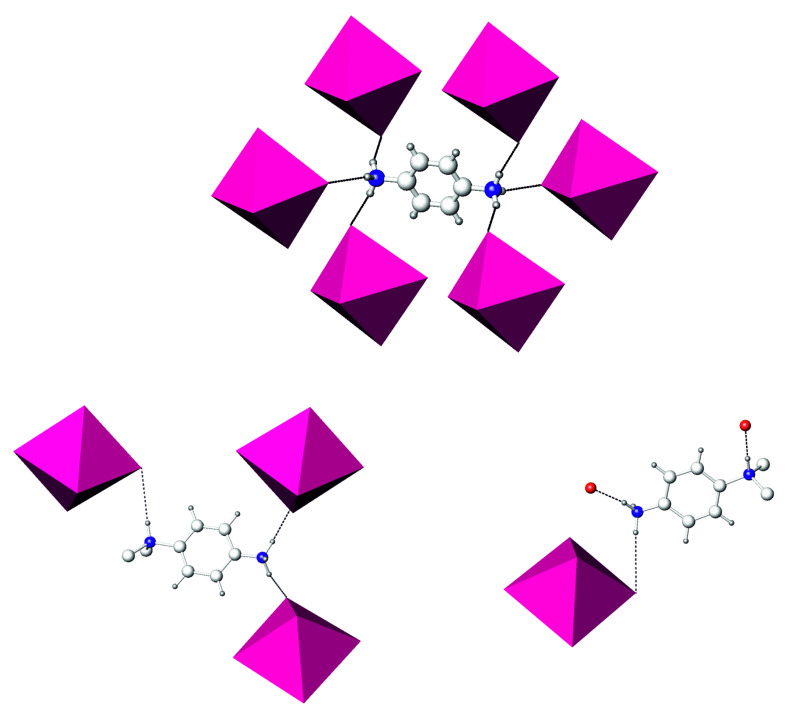
Local coordination of diammonium cations in the crystal structures of **3** (**top**) and **4** (**bottom**). [BiI_6_] octahedra, purple; oxygen, red; nitrogen, blue; carbon, light gray; hydrogen, dark gray. Hydrogen (N)H⋅⋅⋅O and (N)H⋅⋅⋅I bonds are shown as dashed lines. Not all hydrogen atoms are shown for clarity.

The role of the organic dication was also different in the structures of **3** and **4.** In the former structure, the PDA cation used all six hydrogen atoms of two NH_3_-groups to form (N)H⋅⋅⋅I bonds. The H⋅⋅⋅I distances covered the range of 2.72−2.85 Å, which was comparable to those in the crystal structures of **1** and **2**, and linked the layers composed of [BiI_4_] chains and I_2_ molecules into a 3D structure (Figure 6). The DMPDA cation in **4** exploited only three hydrogen atoms out of four available for (N)H⋅⋅⋅I hydrogen bonding. Of the two independent DMPDA cations, one used two hydrogen atoms of the NH_3_-group and a single hydrogen atom of the (CH_3_)_2_NH-group to form (N)H⋅⋅⋅I bonds with d(H⋅⋅⋅I) lengths ranging from 2.66 to 2.88 Å. The second DMPDA cation also formed three hydrogen bonds, one (N)H⋅⋅⋅I with d(H⋅⋅⋅I) = 2.80 Å and two (N)H⋅⋅⋅O bonds with water molecules with H⋅⋅⋅O distances of 1.86 and 1.96 Å, the latter being slightly longer than in similar systems [22]. Therefore, unlike in **3**, the DMPDA cations in the structure of **4** did not saturate their capacity for forming hydrogen bonds. Together with weaker I⋅⋅⋅I noncovalent bonds, this might be a reason for the reduced stability of the latter compound towards hydrolytic decomposition.

**Figure 7:**
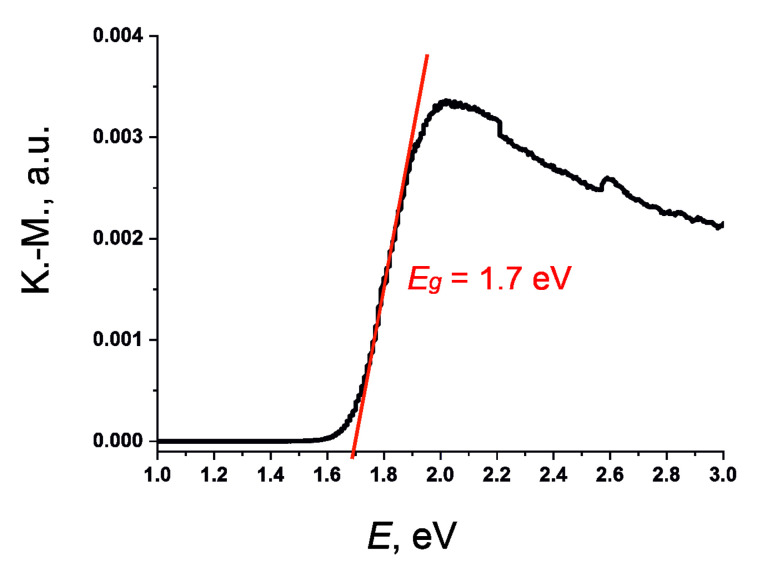
Kubelka–Munk plot for compound **4**.

Compound **4** was black in bulk, but became dark red after grinding into a fine powder. The color nicely corresponds to the optical band gap of 1.7 eV assessed by optical absorption measurements (Figure 7). Comparison with other iodobismuthates reveals that most of those display a band gap near 2 eV, unless polyiodide units are present, narrowing the band gap down to the *E_g_* values of 1.2–1.7 eV. In particular, compound **3** had a band gap of 1.45 eV [14]. The role of the I_2_ and/or I_3_^-^ moieties in diminishing the gap width stems from the band structure of iodobismuthates [41], where the charge transfer occurs from iodine 5*p* states at the top of the valence band to bismuth 6*p* states at the bottom of the conduction band. If I_2_ or I_3_^−^ units are present, they contribute to the very top of the valence band, thus making the band gap narrower or even forming pseudo-localized in-gap states [11]. Depending on the nature of this unit and its position in the crystal structure, the energy difference can vary in a range of 0.5–0.8 eV, frequently leading to a band gap below 1.5 eV [12,13,30]. Therefore, the insertion of I_2_ or I_3_^−^ units can be used as a tool for band gap engineering when attempting to prepare lead-free light harvesting materials based on iodobismuthates.

## 3. Materials and Methods

### 3.1. Synthesis

The starting materials used were Bi (granules, 99.99%), I_2_ (analytical grade), phenylenediamine (analytical grade), *N*,*N*-dimethylphenylenediammonium chloride (analytical grade), P (pure), ethanol (96%), and H_2_O (distilled). BiI_3_ was synthesized from the elements, and hydroiodic acid (stabilized) was synthesized by hydrolysis of freshly prepared PI_3_; details of these procedures are described elsewhere [41]. The HI acid (stabilized) was distilled at 126 °C. Upon exposure to sunlight, the solution acquired a light yellow color owing to the generation of I^3−^ anions.

For the preparation of compound **1**, 5 mL of concentrated HI was added to 0.5 g of phenylenediamine. The solution immediately turned dark brown. The flask with the resulting solution was evaporated in open air for ten days to yield black crystals, which were filtered off and dried on a filter paper. Compound **1** was stable in air for 12 hours, and for several days in a tightly closed vessel.

Columnar well shaped crystals of compound **2** were precipitated from the N,N-dimethylphenylenediammonium chloride solution in a long-term stored 27 wt.% HI acid in four days. The initial light yellow solution gradually turned deep brown, followed by the formation of brown crystals. These crystals were separated by filtration and dried at room temperature.

Compound **3** was synthesized strictly following the literature procedure [14].

Compound **4** was synthesized from the mixture of bismuth triiodide, N,N-dimethylphenylenediammonium chloride, and iodine (at a ratio of 1:2:4). Initial substances were dissolved in 2 mL concentrated HI with 10 mL ethanol forming a dark red solution, which was kept for a week. Brown crystals were separated by filtration under vacuum and dried at room temperature. Compound **4** was stable in open air for several days.

### 3.2. Thermal Analysis

Thermogravimetric analysis was performed using a NETZSCH 209 F1 Libra thermobalance. Samples were heated in alumina crucibles under dry nitrogen flow (20 mL min^−1^) up to 623 or 673 K at a heating rate of 10 K min^-1^. Calibration was performed with CaC_2_O_4_·2H_2_O to ensure accuracy of mass detection of not less than 0.1%. The NETZSCH Proteus Thermal Analysis program was used for data processing.

### 3.3. Powder X-ray Diffraction Analysis (PXRD)

Was performed on an Imaging Plate Guinier Camera (Huber G670, Cu-Kα1 radiation, λ = 1.540598 Å) with 2θ ranging from 3° to 100° at a 0.005° increment. For data collection, crystals were finely crushed in an agate mortar, and the resulting powder was fixed onto a holder using scotch tape.

### 3.4. Crystal Structure Determination

Well-shaped single crystals of **1, 2,** and **4** were selected from the respective synthetic samples.

Single crystal diffraction data of **1** were measured at 100(2) K on a Bruker D8 VENTURE equipped with a PHOTON 100 CMOS detector system and a Mo-target X-ray tube (0.71073 Å). A frame width of 0.50° and an exposure time of 15 *s*/*frame* were employed for data collection. Data reduction and integration were performed with the Bruker software package SAINT (Version 8.37A) [42]. Data were corrected for absorption effects using semi-empirical methods (multi-scan) as implemented in SADABS (Version 2014/5) [43] for **1**. The crystal structures were solved by intrinsic phase methods using the SHELXT (version *2018*/*2*) program package [44], which gave the positions of iodine, nitrogen, carbon, and oxygen atoms. These solutions were refined through full-matrix least-squares procedures using the SHELXL program (version *2018*/*3*) [45]. Positions of hydrogen atoms were found via successive difference. The crystal structure was refined by anisotropic approximations of atomic displacement parameters for all atoms except hydrogens. All H-atoms affiliated to carbons were included at calculated positions and refined as riders, with *U*_iso_(H) = 1.2 *U*_eq_. The H–O bond distances were fixed to 0.99 Å with a standard uncertainty of 0.02 Å for water molecules while both *U*_iso_(H) = 1.5 *U*_eq_(O1). Three H–N1 bond distances were fixed to 0.87 Å with a standard uncertainty of 0.02 Å while *U*_iso_(H) = 1.2 *U*_eq_(N1). Three H–N2 bond distances were restrained to be equal with a standard uncertainty of 0.02 Å while *U*_iso_(H) = 1.2 *U*_eq_(N2). The crystal structure was refined as an inversion twin with the BASF value refined to 0.18(3).

The X-ray diffraction data for single crystals of **2** were collected at 298 K using a STOE STADI VARY diffractometer equipped with a Pilatus100K detector using a rotation method, a collimating mirror, and Mo Kα (0.71073 Å) radiation. STOE X-AREA software was used for cell refinement and data reduction. Data collection and image processing were performed with X-Area 1.67 (STOE&Cie GmbH, Darmstadt, Germany, 2013). The intensity data were scaled up with LANA (part of X-Area) to minimize differences of intensities of symmetry-equivalent reflections (multi-scan method). The structures were solved and refined with the SHELX program [44]. The non-hydrogen atoms were refined by using the anisotropic full matrix least-square procedure. The hydrogen atoms of the cations were calculated and further refined. For this, three H–N1 bond distances were fixed to 0.89 Å with a standard uncertainty of 0.02 Å, one H–N2 bond distance was fixed to 0.98 Å, and *U*_iso_(H) were set as 1.2 *U*_eq_(N) in both cases.

Single crystal diffraction data for compound **4** were measured on a CAD4 diffractometer equipped with an Ag Kα X-ray tube. The experimental data were corrected for Lorenz and polarization factors and absorption effect. The structure was solved by direct methods (*SIR2002* program package) [46]. The solution of the crystal structure revealed positions of bismuth and iodine atoms. Least-squares cycles and successive Fourier synthesis (JANA 2000 [47]) revealed carbon and nitrogen atoms of two independent organic cations and additional separate atoms, which were interpreted as water oxygens. Hydrogen atoms near carbons of aromatic rings were placed geometrically and refined constrained in a riding mode Difference Fourier synthesis in the vicinity of tertiary nitrogen atoms N12 and N22 showed residual peaks at distances corresponding to hydrogen atoms, which were freely refined. Hydrogen atoms near CH_3_ groups were not found, possibly due to their positional disorder; since their location was not critical for understanding the structure, we did not include them in consideration. In the case of NH_3_ groups it was possible to refine hydrogen atoms using a rigid body model.

The summary of experimental and crystallographic information for compounds **1**, **2**, and **4** is given in Table 3. Selected interatomic distances and hydrogen bonding are shown in Table 1 and Table 2, respectively. Further details of the crystal structures may be obtained from Cambridge Crystallographic Data Centre by quoting the CCDC numbers 2101503, 2101502, and 2,101,501 for **1**, **2**, and **4**, respectively. Comparison of the experimental X-ray diffraction patterns with those calculated from the crystal data for **1**, **2**, and **4** are given in Figures S4, S5, and S6 of Supporting Information, respectively.

### 3.5. Raman Spectroscopy

Raman spectra were recorded on a Renishaw In Via spectrometer with laser wavelength of λ = 514 nm (Ar, 50 mW). Sample investigations were performed in backscattering geometry mode using a confocal microscope Leica DMLM (100´ lens) at room temperature in air, with capacity varied via ND (neutral density) filters in the range of 0.0005–15%. Focus distance was 250 mm, and the size of the laser beam was 20 µm. The CCD camera (1024 × 368 pixels) was used as a detector. Scale calibration was conducted using monocrystalline silica (521.5 cm^−1^) as a standard sample. WiRE 3.4 software was used for data processing.

### 3.6. Optical Spectroscopy

Optical diffuse reflectance spectra were recorded using a UV–vis spectrometer Perkin-Elmer Lambda 950 (Perkin-Elmer, Waltham, MA, US) with an attached diffuse reflectance accessory. Measurements were performed at 298 K in the spectral range of 250–1200 nm, with a scanning rate of 2 nm/s using finely ground polycrystalline samples. The data were transformed into absorbance using the Kubelka–Munk method and plotted as [(*k*/*s*)·*h*υ]^2^ against hυ, where *k* is the absorption coefficient, *s* is the scattering coefficient, and *h* is the Planck constant [48,49]. Optical band gap, *Eg*, was approximated by extrapolation to *k* = 0.

## 4. Conclusions

We found that dications of phenylenediamine and its N,N-dimethyl derivative, PDA and DMPDA respectively, reacted with iodine-enriched hydroiodic acid to form triiodides **1** and **2**. If a source of bismuth was added, hybrid polyiodobismuthates **3** and **4** were formed. The PDA and DMPDA polyiodobismuthates featured band gaps of 1.45 and 1.7 eV, respectively, within the optimal range for efficient solar light absorbers. In all four crystal structures, the diammonium cations provided linking of the respective anionic parts into polymeric structures with the help of (N)H⋅⋅⋅I intermolecular bonds. The interatomic H⋅⋅⋅I distances ranged from 2.66 to 2.88 Å, indicating that the intermolecular bonds were relatively strong. Additionally, in the structures of **1** and **4**, the dications formed hydrogen (N)H⋅⋅⋅O bonds with water of crystallization. However, the bonding ability of the dications was different in different structures, which manifested in a different number of hydrogen atoms used to form (N)H⋅⋅⋅I and (N)H⋅⋅⋅O bonds, from three in **4** to six in **3**.

Further interaction within the inorganic substructures was provided by intermolecular I⋅⋅⋅I bonds of different natures. In **3**, the [BiI_6_]⋅⋅⋅I_2_ bonds were rather short; their length of 3.53 Å was comparable with the intralayer I⋅⋅⋅I bonds in the crystal structure of elemental iodine (3.50 Å). More typical were longer interatomic distances, ranging from 3.76 to 4.07 Å, which could be found in **1**, **3**, and **4**. Unlike in the structure of **1**, where the I⋅⋅⋅I non-covalent bonds ensured linking the I_3_^-^ anions into chains, compounds **3** and **4** displayed interactions of iodine atoms in the vertices of the [BiI_6_] octahedra. Although the I⋅⋅⋅I intermolecular distances were rather long, they were found to affect the band structure of iodobismuthates leading to narrowing of the band gap, although to a lesser extent than the secondary I⋅⋅⋅I bonds.

In general, various intermolecular interactions ensure the formation of supramolecular architectures from simple organic and inorganic blocks. Although a single intermolecular interaction might be weak, a combination of various weak forces acting within an organic–inorganic system leads to improved stability of a resulting hybrid. Given the stability of the building blocks in a strong acidic solution, a strategy of converting bismuth and other post-transition metals into respective iodometallates and polyiodometallates can be developed aiming at the facile synthesis of hybrid lead-free light-harvesting materials.

## Figures and Tables

**Table 1:** Selected Interatomic Distances and Angles in the Anionic Part of the Crystal Structures of **1**, **2**, and **4**.

Atoms	Distance, Å	Atoms	Angle, °
**1**			
I1–I2 I1–I5 I3–I4 I3–I6 I2···I5^i^ I4···I6^i^	2.9051(7) 2.9603(7) 2.9212(7) 2.9464(7) 3.7702(9) 3.7557(9)	I2—I1—I5 I4—I3—I6	176.880(13) 177.302(13)
**2**			
I2–I3	2.9966(10)	I3—I2—I4	178.13(3)
I2–I4	2.8566(11)		
**4**			
Bi1–I1 Bi1–I2 Bi1–I3 Bi1–I4 Bi1–I5 Bi1–I6 I7–I8 I9–I10 I5···I10	3.0113(9) 3.0818(9) 3.1877(9) 3.0374(9) 3.0729(10) 3.0878(9) 2.9222(16) × 2 2.951(4) × 2 3.965(2)	I1—Bi1—I2 I2—Bi1—I3 I1—Bi1—I3 I2—Bi1—I5 I4—Bi1—I6 I8—I7—I8 I10—I9—I10	92.32(3) 90.59(3) 177.02(3) 174.85(3) 179.46(3) 180 180

Symmetry codes: (i) *x* − 1/2, − *y* + 1/2, *z*.

**Table 2:** Hydrogen Bonding in the Crystal Structures of **1**, **2**, and **4**.

D–H···A	d(D–H), Å	d(H···A), Å		d(D···A), Å	Angle (D–H···A), °
	**1**				
N1–H1A···O1	0.94(2)	1.99(2)		2.926(4)	171(4)
N1–H1B···I6	0.89(2)	2.75(2)		3.640(3)	176(4)
N2–H2B···I5	0.92(3)	2.75(3)		3.621(3)	158(4)
N2–H2C···O1	0.92(3)	2.03(3)		2.946(4)	172(4)
O1–H1E···I2	0.95(2)	2.84(4)		3.593(3)	137(4)
	**2**				
N1–H1A···I3	0.89	2.79		3.554(9)	144.4
N1–H1B···I1	0.89	2.63		3.491(8)	163.7
N1–H1C···I1	0.89	2.60		3.492(9)	177.7
N2–H2···I1	0.98	2.54		3.506(11)	168
	**4**				
N22–H22n···O2	0.920(14)	1.862(11)		2.780(17)	175.7(8)
N1ba–H3ba···I3	0.96	2.79		3.7423(12)	167.56(2)
N1ba–H1ba···O1	0.96	1.96		2.911(7)	168.792(16)
N12–H12n···I3	0.920(13)	2.6597(8)		3.57(13)	174.45(7)
N1ra–H3ra···I2	0.96	2.88		3.5333(10)	126.07(2)
N1ra–H2ra···I6	0.96	2.77		3.5934(10)	143.20(2)

**Table 3:** Crystallographic data for **1**, **2**, and **4**.

Compound	1	2	4
Empirical formula	C_6_H_12_I_6_N_2_O	C_8_H_14_I_4_N_2_	C_32_H_40_Bi_2_I_18_N_8_O_3_
Formula weight	889.58	645.81	3287
Temperature (K)	100(2)	293(2)	295(2)
Wavelength (Ǻ)	0.71073	1.54186	0.56083
Crystal system	Orthorhombic	Monoclinic	Monoclinic
Space group	*Pna*2_1_	*P*2_1_/n	*C*2/c
*a* (Å)	19.080(4)	9.3708(2)	20.203(3)
*b* (Å)	7.9300(16)	18.1996(4)	18.239(3)
*c* (Å)	11.469(2)	9.8916(2)	21.018(4)
*α* (°)	90	90	90
*β* (°)	90	99.818(2)	100.263(14)
*γ* (°)	90	90	90
*V* (Å^3^)	1735.3(6)	1662.25(6)	7621(2)
*Z*	4	4	4
*ρ*_calcd_ (g·cm^−3^)	3.405	2.581	2.8639
*μ* (mm^−1^)	10.725	58.685	6.405
*F*(000)	1552	1152	5696
Crystal size (mm)	0.12 × 0.15 × 0.22	0.10 × 0.15 × 0.20	0.31 × 0.27 × 0.15
*θ* range for data collection (°)	3.123–41.198	4.860–73.002	2.03-20.97
Reflections collected	131330	24943	8595
Independent reflections	11518 [*R*_int_ = 0.0307]	3310 [*R*_int_ = 0.112]	4724 [*R*_int_ = 0.0132]
Data/restraints/params.	11518/9/161	3310/0/132	4724/0/289
*R*1, *wR*2 *(I* > 2*σ*(*I*))	0.0194, 0.0354	0.0486, 0.1570	0.406, 0.567
*R*1, *wR*2 (all data)	0.0245, 0.0366	0.0545, 0.1621	0.406, 0.567
*Δρ*_max_ (e/Å^−3^) positive/negative	1.211/−1.200	0.867/−0.809	0.57/−0.64
Goodness-of-fit	1.170	1.130	1.15

## Supplementary Materials

The following are available online, Figure S1: Thermal analysis data for **1**, Figure S2: Thermal analysis data for **2**, Figure S3: Thermal analysis data for **4**, Figure S4: X-ray powder diffraction pattern for **1**, Figure S5: X-ray powder diffraction pattern for **2**, and Figure S6: X-ray powder diffraction pattern for **4**.

## Sample Availability

Samples of the compounds **1**, **2, 4** are available from the authors.

## References

[1] Petrov A.A., Tarasov A.B. (2020). Methylammonium polyiodides in perovskite photovoltaics: From fundamentals to applications. Front. Chem..

[2] Petrov A.A., Belich N.A., Grishko A.Y., Stepanov N.M., Dorofeev S.G., Maksimov E.G., Shevelkov A.V., Zakeeruddin S.M., Grätzel M., Tarasov A.B. (2017). New formation strategy of hybrid perovskites via room temperature reactive polyiodide melts. Mater. Horizons..

[3] Turkevych I., Kazaoui S., Belich N.A., Grishko A.Y., Fateev S.A., Petrov A.A., Urano T., Aramaki S., Kosar S., Kondo M. (2019). Strategic advantages of reactive polyiodide melts for scalable perovskite photovoltaics. Nat. Nanotech..

[4] Belich N.A., Petrov A.A., Rudnev P.O., Stepanov N.M., Turkevych I., Goodilin E.A., Tarasov A.B. (2020). From metallic lead films to perovskite solar cells through lead conversion with polyhalides solutions. ACS Appl. Mater. Interfaces.

[5] Lu L., Pan X., Luo J., Sun Z. (2020). Recent Advances and Optoelectronic Applications of Lead-Free Halide Double Perovskites. Chem. Eur. J..

[6] Adonin S.A., Sokolov M.N., Fedin V.P. (2018). Polyhalide-bonded metal complexes: Structural diversity in an eclectic class of compounds. Coord. Chem. Rev..

[7] Xiao Z., Song Z., Yan Y. (2019). From Lead Halide Perovskites to Lead-Free Metal Halide Perovskites and Perovskite Derivatives. Adv. Mater..

[8] Fakharuddin A., Shabbir U., Qiu W., Iqbal T., Sultan M., Heremans P., Schmidt-Mende L. (2019). Inorganic and Layered Perovskites for Optoelectronic Devices. Adv. Mater..

[9] Ozório M.S., Oliveira W.X.C., Silveira J.F.R.V., Nogueira A.F., Da Silva J.L.F. (2020). Novel zero-dimensional lead-free bismuth based perovskites: From synthesis to structural and optoelectronic characterization. Mater. Adv..

[10] Starkholm A., Kloo L., Svensson P.H. (2019). Polyiodide hybrid perovskites: A strategy to convert intrinsic 2D systems into 3D photovoltaic materials. ACS Appl. Energy Mater..

[11] Zhang W., Liu X., Li L., Sun Z., Han S., Wu Z., Luo J. (2018). Triiodide-induced band-edge reconstruction of a lead-free perovskite-derivative hybrid for strong light absorption. Chem. Mater..

[12] Zhang W., Kou B., Peng Y., Wu Z., Yao Y., Dey D., Lia L., Luo J. (2018). Rational design of a triiodide-intercalated dielectric-switching hybrid for visible-light absorption. J. Mater. Chem. C.

[13] Shestimerova T.A., Yelavik N.A., Mironov A.V., Kuznetsov A.N., Bykov M.A., Grigorieva A.V., Shevelkov A.V. (2018). From Isolated Anions to Polymer Structures through Linking with I_2_: Synthesis, Structure, and Properties of Two Complex Bismuth(III) Iodine Iodides. Inorg. Chem..

[14] Shestimerova T.A., Golubev N.A., Yelavik N.A., Bykov M.A., Grigorieva A.V., Wei Z., Dikarev E.V., Shevelkov A.V. (2018). Role of I_2_ Molecules and Weak Interactions in Supramolecular Assembling of Pseudo-Three-Dimensional Hybrid Bismuth Polyiodides: Synthesis, Structure, and Optical Properties of Phenylenediammonium Polyiodobismuthate(III). Cryst. Growth Design.

[15] Kloo L., Rosdahl J., Svensson P.H. (2002). On the intra- and intermolecular bonding in polyiodides. Eur. J. Inorg. Chem..

[16] Svensson P.H., Kloo L. (2003). Synthesis, Structure, and Bonding in Polyiodide and Metal Iodide−Iodine Systems. Chem. Rev..

[17] Hagfeldt A., Boschloo G., Sun L., Kloo L., Pettersson H. (2010). Dye-Sensitized Solar Cells. Chem. Rev..

[18] Savastano M. (2021). Words in supramolecular chemistry: The ineffable advances of polyiodide chemistry. Dalton Trans..

[19] Williams J.M., Wang H.H., Emge T.J., Geiser U., Beno M.A., Leung P.C.W., Carlson K.D., Thorn R.J., Schultz A.J. (1987). Rational design of synthetic metal superconductors. Prog. Inorg. Chem..

[20] Shestimerova T.A., Bykov M.A., Wei Z., Dikarev E.V., Shevelkov A.V. (2019). Crystal structure and two-level supramolecular organization of glycinium triiodide. Russ. Chem. Bull. Int. Ed..

[21] Savinkina E.V., Golubev D.V., Grigoriev M.S. (2019). Synthesis, characterization, and crystal structures of iodides and polyiodides of scandium complexes with urea and acetamide. J. Coord. Chem..

[22] Mezentsev-Cherkes I.A., Shestimerova T.A., Medved’ko A.V., Kalinin M.A., Kuznetsov A.N., Wei Z., Dikarev E.V., Vatsadze S.Z., Shevelkov A.V. (2021). Synthesis and supramolecular organization of the iodide and triiodides of a polycyclic adamantane-based diammonium cation: The effects of hydrogen bonds and weak I⋯I interactions. CrystEngComm.

[23] Peuronen A., Rinta H., Lahtinen M. (2015). N···I halogen bonding supported stabilization of a discrete pseudo-linear [I_12_]^2^^-^ polyiodide. CrystEngComm.

[24] Li T., Hu Y., Morrison C.A., Wu W., Hana H., Robertson N. (2017). Lead-free pseudo-three-dimensional organic–inorganic iodobismuthates for photovoltaic applications. Sustain. Energy Fuels.

[25] Nesterova O.V., Petrusenko S.R., Dyakonenko V.V., Shishkin O.V., Linert W. (2006). A three-dimensional framework of bis [tris(ethylenediamine)zinc] tetraiodocadmate diiodide assisted by N—H…I hydrogen bonds. Acta Crystallogr. C.

[26] Shestimerova T.A., Mironov A.V., Bykov M.A., Grigorieva A.V., Wei Z., Dikarev E.V., Shevelkov A.V. (2020). Assembling Polyiodides and Iodobismuthates Using a Template Effect of a Cyclic Diammonium Cation and Formation of a Low-Gap Hybrid Iodobismuthate with High Thermal Stability. Molecules.

[27] Yushina I.D., Batalov V.I., Bartashevich E.V., Davydov A.O., Zelenovskiy P.S., Masunov A.E. (2017). Raman spectroscopy and theoretic study of hyperpolarizability effect in diiodobutenyl-bis-thioquinolinium triiodide at low temperature. J. Raman Spectrosc..

[28] Yushina I.D., Tarasova N.M., Kim D.G., Sharutin V.V., Bartashevich E.V. (2019). Noncovalent bonds, spectral and thermal properties of substituted thiazolo [2, 3-b][1, 3] thiazinium triiodides. Crystals.

[29] Deplano P., Ferraro J.R., Mercuri M.L., Trogu E.F. (1999). Structural and Raman spectroscopic studies as complementary tools in elucidating the nature of the bonding in polyiodides and in donor-I_2_ adducts. Coord. Chem. Rev..

[30] Adonin S.A., Usoltsev A.N., Novikov A.S., Kolesov B.A., Fedin V.P., Sokolov M.N. (2020). One- And Two-Dimensional Iodine-Rich Iodobismuthate(III) Complexes: Structure, Optical Properties, and Features of Halogen Bonding in the Solid State. Inorg. Chem..

[31] Yelovik N.A., Shestimerova T.A., Bykov M.A., Wei Z., Dikarev E.V., Shevelkov A.V. (2017). Synthesis, structure, and properties of LnBiI_6_⋅13H_2_O (Ln = La, Nd). Russ. Chem. Bull. Int. Ed..

[32] Chang J.-H., Albrecht R., Doert T., Ruck M. (2019). The Water-Rich Iodidobismuthate (H_3_O)Rb_3_BiI_7_⋅4H_2_O. Z. Anorg. Allg. Chem..

[33] Bi W., Louvain N., Mercier N., Luc J., Sahraoui B. (2007). Type structure, which is composed of organic diammonium, triiodide and hexaiodobismuthate, varies according to different structures of incorporated cations. CrystEngComm.

[34] Dehnhardt N., Luy J.-N., Szabo M., Wende M., Tonner R., Heine J. (2019). Synthesis of a two-dimensional organic–inorganic bismuth iodide metalate through in situ formation of iminium cations. Chem. Commun..

[35] Adonin S.A., Gorokh I.D., Novikov A.S., Samsonenko D.G., Yushina I.V., Sokolov M.N., Fedin V.P. (2018). Halobismuthates with halopyridinium cations: Appearance or non-appearance of unusual colouring. CrystEngComm.

[36] Dennington A.J., Weller M.T. (2016). Synthesis and structure of pseudo-three-dimensional hybrid iodobismuthate semiconductors. Dalton Trans..

[37] Bartashevich E.V., Tsirelson V.G. (2014). Interplay between non-covalent interactions in complexes and crystals with halogen bonds. Russ. Chem Rev..

[38] Alvarez S. (2013). A cartography of the van der Waals territories. Dalton Trans..

[39] Pyykko P. (1997). Strong Closed-Shell Interactions in Inorganic Chemistry. Chem. Rev..

[40] Chernyshov I.Y., Ananyev I.V., Pidko E.A. (2020). Revisiting van der Waals Radii: From Comprehensive Structural Analysis to Knowledge-Based Classification of Interatomic Contacts. ChemPhysChem.

[41] Yelovik N.A., Mironov A.V., Bykov M.A., Kuznetsov A.N., Grigorieva A.V., Wei Z., Dikarev E.V., Shevelkov A.V. (2016). Iodobismuthates Containing One-Dimensional BiI_4_^–^ Anions as Prospective Light-Harvesting Materials: Synthesis, Crystal and Electronic Structure, and Optical Properties. Inorg. Chem..

[42] (2019). SAINT, Version 8.38A.

[43] Krause L., Herbst-Irmer R., Sheldrick G.M., Stalke D. (2015). Comparison of silver and molybdenum microfocus X-ray sources for single-crystal structure determination. J. Appl. Cryst..

[44] Sheldrick G.M. (2015). SHELXT–Integrated space-group and crystal-structure determination. Acta Cryst..

[45] Sheldrick G.M. (2015). Crystal structure refinement with SHELXL. Acta Cryst..

[46] Burla M.C., Camalli M., Carrozzini B., Cascarano G., Giacovazzo C., Polidori G., Spagna R. (2003). SIR2002: The program. J. Appl. Cryst..

[47] Petricek V., Dusek M., Palatinus L. (2000). Jana2000. Structure Determination Software Programs.

[48] Kubelka P., Munk F. (1931). Ein Beitrag zur Optik der Farbanstriche (Contribution to the optic of paint). Z. Tech. Phys..

[49] Fedeli P., Gazza F., Calestani D., Ferro P., Besagni T., Zappettini A., Calestani G., Marchi E., Ceroni P., Mosca R. (2015). Influence of the synthetic procedures on the structural and optical properties of mixed-halide (Br, I) perovskite films. J. Phys. Chem. C.

