# Supplementary material for: Molecular and Supramolecular Structures of Triiodides and Polyiodobismuthates of Phenylenediammonium and Its N,N-dimethyl Derivative
Source: Molecules. 2021 Sep 21;26(18):5712. doi: 10.3390/molecules26185712 (PMC8470886; doi:10.3390/molecules26185712)
Supplement: Supplementary file 1 [file molecules-26-05712-s001.zip › molecules-1365891-supplementary.pdf]

Molecular and Supramolecular Structures of Triiodides and Polyiodobismuthates of Phenylenediammonium and Its N,N-dimethyl Derivative

Tatiana A. Shestimerova,[1] Nikita A. Golubev,[1] Mikhail A. Bykov,[1] Andrei V. Mironov,[1] Sergey A. Fateev, [2] Alexey B. Tarasov, [1,2] Ivan Turkevych,[3] Zheng Wei,[4] Evgeny V. Dikarev,[4] and Andrei V. Shevelkov[1,*]

[1] Department of Chemistry, Lomonosov Moscow State University, 119991 Moscow, Russia,
[2] Department of Materials Science, Lomonosov Moscow State University, 119991 Moscow, Russia
[3] Sensing System Research Center, National Institute of Advanced Industrial Science and Technology, 305-8565 Tsukuba, Japan
[4] Department of Chemistry, University at Albany, SUNY, Albany New York 12222, United States
* Correspondence: shev@inorg.chem.msu.ru;

Table of Contents

Figure S1. Thermal analysis data for **1**

Figure S2. Thermal analysis data for **2**

Figure S3. Thermal analysis data for **4**

Figure S4. X-ray powder diffraction pattern for **1**

Figure S5. X-ray powder diffraction pattern for **2**

Figure S6. X-ray powder diffraction pattern for **4**

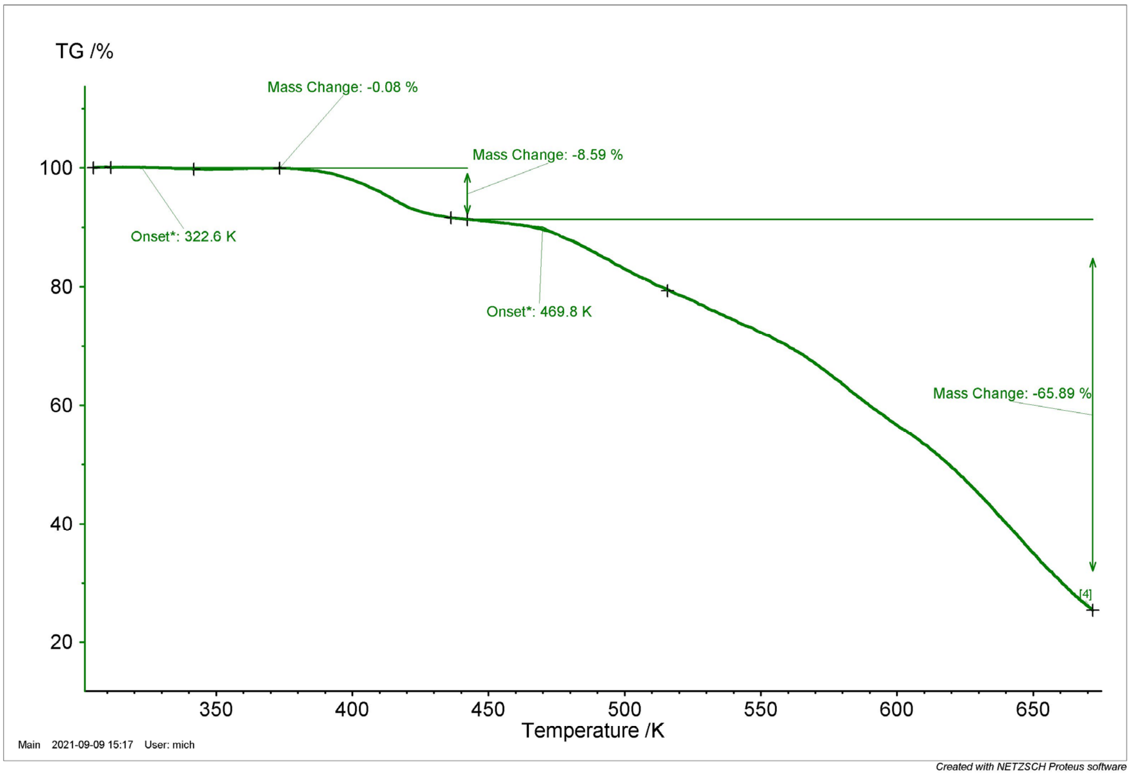

Figure S1. Thermal analysis data for **1**. Compound **1** decomposes in two steps. Near 310 K it loses 27.41 %, which corresponds to one I_2 molecule (calc. 24,5 %). At the second stage, near 380 K, it loses another 28.90 %, which can be attributed to one I_2 molecule and one water molecule (calc. 28.0 % in total).

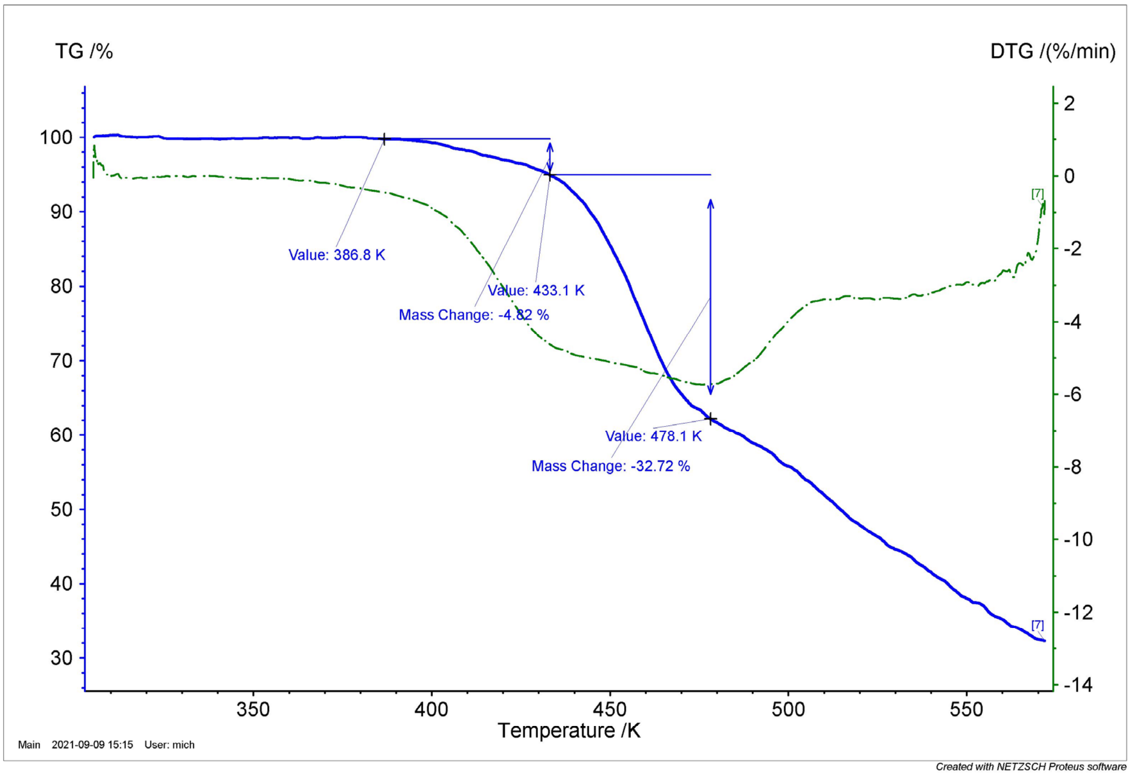

Figure S2. Thermal analysis data for **2**. Compound **2** begins to decompose at 387 K. There are no clearly defined stages of decomposition on the graph, but the total value of the mass loss to the inflection point at 478 K is 37,55 %, which is attributed to a I_2 molecule (calc. 39,3 %).

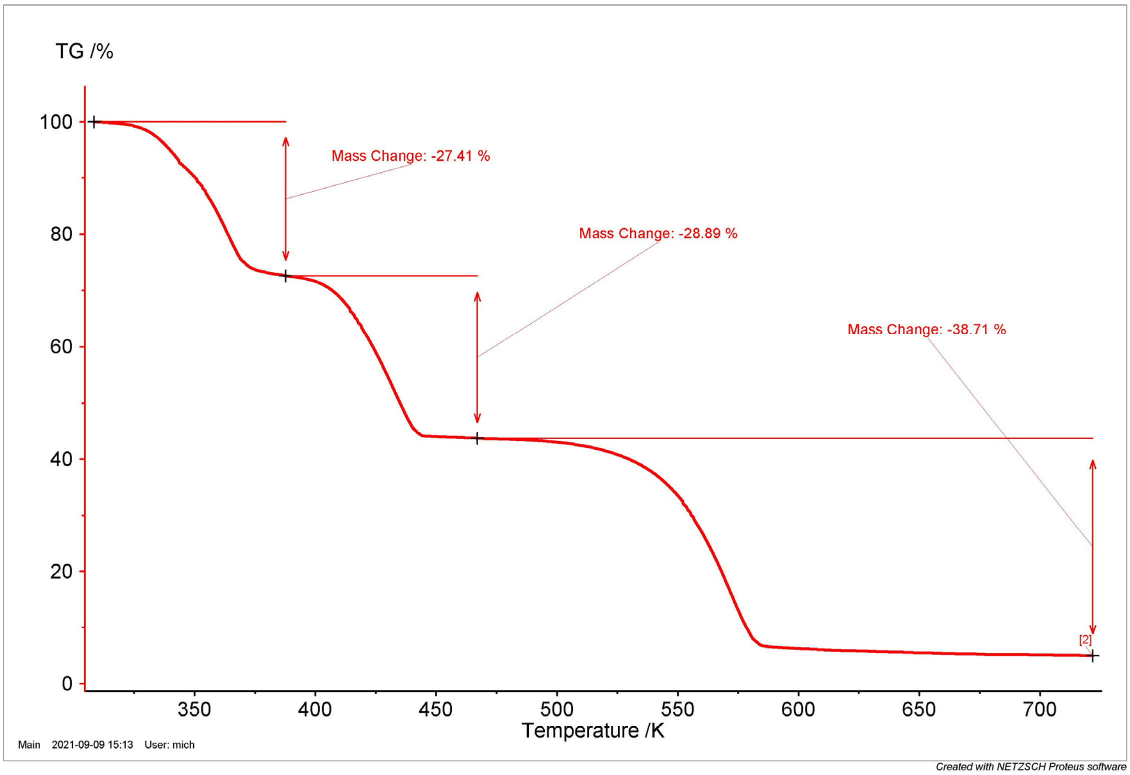

Figure S3. Thermal analysis data for **4**. Compound **4** is stable up to 373 K, then it loses 8.67 % of its mass, which may correspond to the removal of part of I2 and water molecules, so that ½ I_2 and all H_2O (calc. 9.9 % in total) are released.

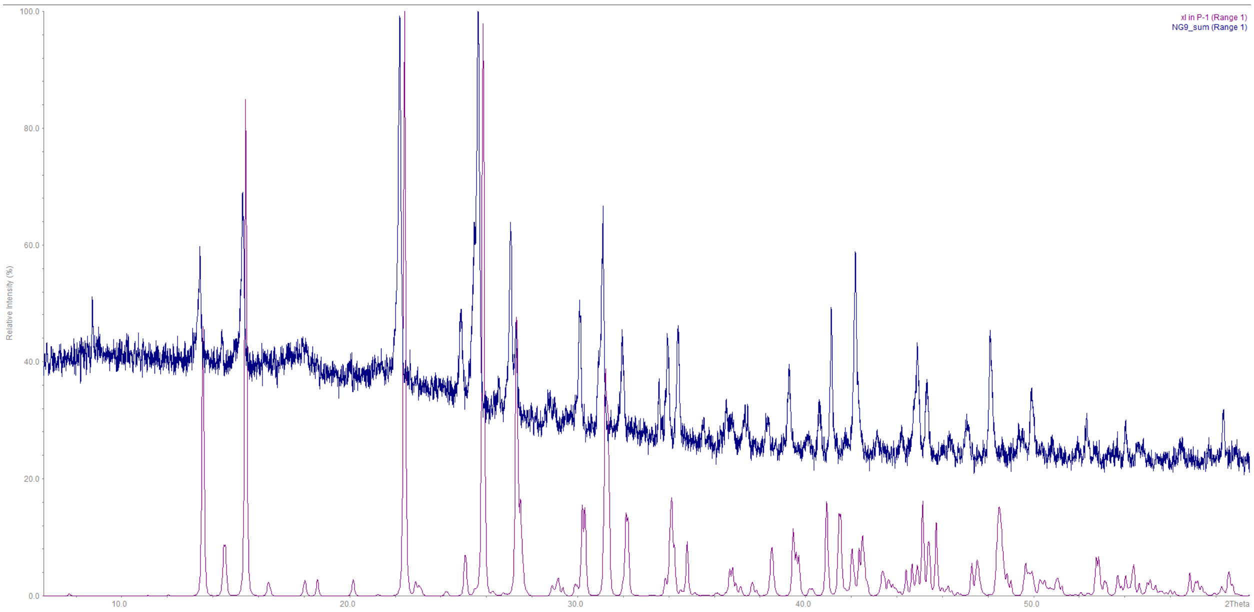

Figure S4. X-ray powder diffraction pattern for **1**. Experimental profile (measured at room temperature), blue; calculated from the crystal data (measured at 100 K), magenta.

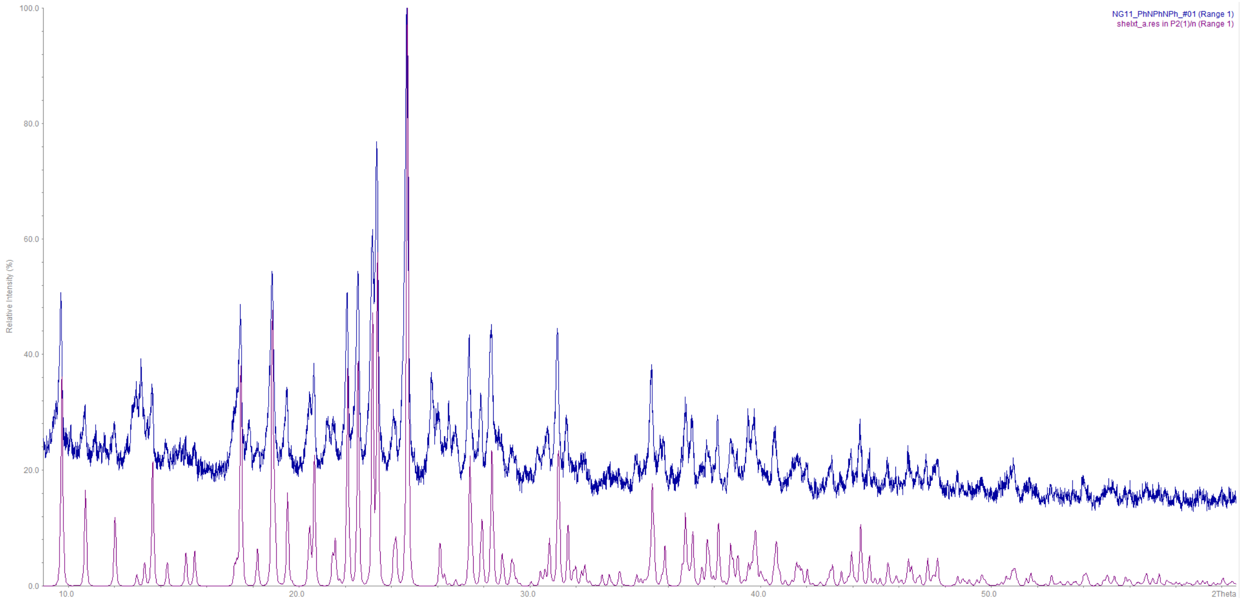

Figure S5. X-ray powder diffraction pattern for **2**. Experimental profile, blue; calculated from the crystal data, magenta.

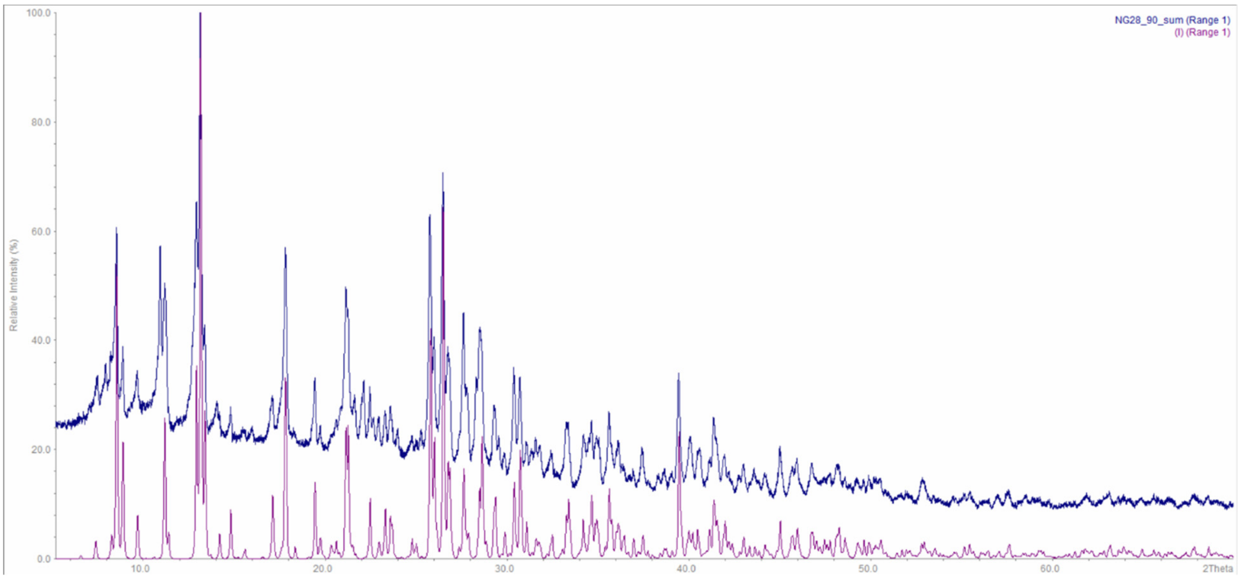

Figure S6. X-ray powder diffraction pattern for **4**. Experimental profile, blue; calculated from the crystal data, magenta.
